# Critically ill COVID-19 patient

**DOI:** 10.3906/sag-2004-122

**Published:** 2020-04-21

**Authors:** Burçin HALAÇLI, Akın KAYA, Arzu TOPELİ

**Affiliations:** 1 Division of Intensive Care Medicine, Department of Internal Medicine, Hacettepe University Faculty of Medicine, Ankara Turkey; 2 Pulmonary and Critical Care, Ankara University Faculty of Medicine, Ankara Turkey

**Keywords:** Coronavirus, SARS-CoV 2, pandemic, respiratory failure, ARDS, intensive care

## Abstract

Coronavirus disease 2019 (COVID-19) stands out as the major pandemic that we have experienced in the last century. As it affects every social structure, it brought the importance of intensive care support once again to the agenda of healthcare system after causing severe acute respiratory syndrome. The precautions to be taken against this virus, where our knowledge is extremely small, intensive care units take an indispensable place in pandemic planning. In this review, we aimed to emphasize the crucial points regarding intensive care management of COVID-19 patients, which we have written not only for intensivists but also for all healthcare professionals.

## 1. Introduction 

The consequences of coronavirus disease 2019 (COVID-19) caused by severe acute respiratory syndrome coronavirus 2 (SARS-CoV 2) which was originated in Wuhan region of China and spread all over the world, have influenced all stages of health services. Eventually, World Health Organization (WHO)^1^1https://www.who.int/emergencies/diseases/novel-coronavirus-2019 has declared pandemic for this virus. This pandemic is accepted as a viral pneumonia pandemic not a simple flu, therefore, intensive care unit (ICU) admission, follow-up, and management of the critically ill patients with COVID-19 is extremely important. Due to the paucity of studies on COVID-19, suggestions are generally within the framework of the experiences gained from China, Italy, USA, and UK that have the biggest war against this disease. In this review, we summarized the essential points of ICU management for COVID-19 patients.^1^

## 2. General characteristics of critically ill COVID-19 patients for ICU admission 

The need for intensive care might differ according to institutions or even countries, depending on the demand and supply ranging from 5% to 32% [1–3]. Severe disease may present with severe acute respiratory infection (SARI) i.e. severe pneumonia and acute respiratory distress syndrome (ARDS) which is reported in 60%–70% of patients; sepsis and septic shock reported in 30%; myocarditis, arrhythmia, and cardiogenic shock in 20%–30%; and acute kidney injury in 10%–30% of patients [4]. Although respiratory failure is often hypoxemic, hypercapnic respiratory failure might also be seen mainly due to mucus plugs. There is a male dominance with male to female ratio being 2/1 in severe cases, whereas according to a recent data from Italy, 82% of patients were male. Although hypertension and diabetes mellitus are the most common reported comorbidities, advanced age is also a risk factor for the development of severe disease [5]. The Main ICU admission indications according to Turkish Guidelines^2^2https://hsgm.saglik.gov.tr/depo/covid19/rehberler/COVID-19_Rehberi.pdf (Turkish Public Health General Directorate Guidelines), which are prepared according to COVID-19 Advisory Committee proposals are shown in Table. Patients who are hemodynamically stable and do not need advanced respiratory and organ support anymore might be transferred to wards [6]. 

**Table 1:** The Main ICU admission indications according to Turkish Guidelines^12^ (Turkish Public Health General Directorate Guidelines)

Patients with respiratory rate ≥ 30
Dyspnoea and increased work of breathing
SpO2 < 90% or < 70 mm Hg (in room air)
Oxygen requirement ≥ 5L/min with nasal cannula
Lactate > 2 mmol/L
Hypotension (systolic blood pressure (SBP) < 90 mmHg, > 40 mmHg drops from usual SBP, mean arterial pressure (MAP) < 65 mmHg)
Skin hypoperfusion signs
Organ dysfunction such as confusion, kidney and liver tests abnormalities, thrombocytopenia, elevated troponin level and arrhythmia

^12^https://hsgm.saglik.gov.tr/depo/covid19/rehberler/COVID-19_Rehberi.pdf

The median time between the initiation of clinical signs to the development of pneumonia is about 5 days and the median ICU admission time after development of hypoxemia is about 7–12 days (1). ICU mortality due to COVID-19 varies from 16% to 78% according to different studies [2,5,7–9]. The current reported mortality rate was 26% in a recent multicentre study from Italy. However, 58% of patients were still in ICU and only 16% of patients were able to be discharged which means mortality rate could be higher [3]. Older age, presence of comorbidities such as hypertension, diabetes, cardiovascular disease, chronic lung disease, cancer, higher d-dimer and C-reactive protein, and lower lymphocyte levels are associated with higher mortality [4]. 

## 3. ICU organization

In part of a pandemic plan of the country and hospitals, ICU beds should be organized as well as all devices, equipment, and personnel. It is appropriate to reserve some ICUs for COVID-19 patients. If this is not possible, patient cohorts should be done within the ICU. Patients should be followed in isolated rooms, necessary personnel protective equipment (PPE) should be provided and a dedicated team for COVID ICU should be organised. For this purpose, patient to nurse ratio could be 1:1, if possible. A stand-by clean medical team may also be considered. This team can work in other ICUs. It is recommended to change the team that takes care of COVID-19 patients in every 14 days. Hereby, a wash-out period is provided. It is also suggested that health care workers should not work for longer than 12 h a day in order to minimise risk of infection. They must be warned that the whole team should report their body temperature and symptoms twice a day and they will not leave the city during rest periods [10]. 

It should be considered that the existing intensive care beds and organization may not be sufficient due to the pandemic. Potential solutions are expanding intensive care outside ICUs, like high-dependency units, general wards, postanaesthesia care units, and even operating rooms. On the other hand, surging in the ICU capacity brings an incremental demand for equipment such as ventilators, consumable materials, therapeutics and as well as human force as healthcare workers (HCW). For this purpose, elective in-patient admissions and surgeries should be delayed and available wards could be adapted for stepping down other ICU patients in order to evacuate ICUs. Another point is to increase HCW trained in intensive care. Therefore, HCW who are currently working out of ICU should be systematically trained in terms of COVID-19 protocol for supporting ICU staff [11]. 

## 4. Infection control 

To ensure proper infection control measures in the ICU are crucial. Contact and droplet isolation precautions should be undertaken^3^3Government of Canada. Infection prevention and control for novel coronavirus (2019-nCoV): interim guidance for acute healthcare settings. https://www.canada.ca/en/public-health/services/diseases/2019-novel-coronavirus- infection/health-professionals/interimguidance- acute-healthcare-settings.html (accessed Februay 2020). Faecal-oral transmission has also been reported^4^4XINHUANET. Novel coronavirus may spread via digestive system: experts. English.news.cn. http:// www.xinhuanet.com/english/2020- 02/02/c_138749620.htm (accessed February 2020). Viral shedding is expected to be extremely high in critically ill patients. Interventions such as bag-valve-mask ventilation, noninvasive mechanical ventilation (NIMV), high flow nasal cannula oxygen (HFNO), nebulisation and oro-tracheal intubation automatically bring high risk in terms of aerosol production. Expanded aerosol production might increase airborne transmission risk as well. That is why airborne isolation (with negative pressure) should ideally be implemented as soon as possible in a single room with negative pressure supply that should be provided at least >12 h/day^5^5WHO. Global surveillance for COVID-19 caused by human infection with COVID-19 virus: interim guidance. https://www. who.int/docs/ default-source/coronaviruse/global-surveillance-for-covid-v-19- nal200321-rev.pdf (accessed March 22, 2020).. Fluid-resistant gown, two longer sleeved gloves to prevent exposure of the wrists with glove subsiding, eye glasses, full face shield, hair covers or hood, N95 mask and shoe worn are recommended for exposure to confirmed or suspected COVID-19 patients. Disposable shoe covers might increase the risk of self-contamination during removal of protection clothing and should be avoided. All ICU team should wear hospital’s scrubs which should not be worn outside the hospital^6^6Government of Canada. Infection prevention and control for novel coronavirus (2019-nCoV): interim guidance for acute healthcare settings. https://www.canada.ca/en/public-health/services/diseases/2019-novel-coronavirus infection/health-professionals/interim-guidance-acutehealthcare- settings.html (accessed February 2020). Zones should be provided for wearing and taking off properly. Powered air purifying respirators (PAPRs) are other protective equipment which has not been proven so far, but it seems reasonable to use in order to decline the viral transmission. It is more convenient for prolonged resuscitations [12]. 

## 5. Respiratory support

Hypoxemic respiratory failure should be recognized early. Despite conventional oxygen therapy, increased work of breathing and hypoxemia could become progressively worse. Supplemental oxygen should be given via low-flow O2 delivery systems such as nasal cannula (1-6 L/min to provide FiO2 of 0.24–0.45), simple face mask (5–8 L/min to provide FiO2 up to 0.50–0.60), nonrebreather masks with reservoir providing FiO2 up to > 0.85 with 10–15 L/min oxygen, titrated according to SpO2. Venturi and diffuser masks are suggested to be avoided. It should be remembered that FiO2 > 0.60 for > 6 h might create O2 toxicity. HFNO therapy and NIMV support may be applied in selected hypoxemic respiratory failure cases with proper PPE because of high risk of aerosol generation. However, these patients should be followed closely in terms of clinical deterioration, if no positive response is obtained in the first few hours (refractory hypoxemia, tachypnoea, tidal volume (Vt) > 9 mL/kg meaning increased minute ventilation and work of breathing). When applying NIMV, a helmet mask may be used, applied with intensive care ventilators or dual-circuit ventilators; a viral/bacterial filter should be added to the circuit. NIMV should not be applied to patients whose secretions cannot be controlled; who have high aspiration risk, impaired mental status, cardiac complications and multiple organ failure; and who are hemodynamically unstable [13]. Prolonged spontaneous breathing may cause uncontrolled intrathoracic negative pressures and induce patient-self-inflicted lung injury similar to ventilator induced lung injury and therefore, must be prevented by utilization of intubation as soon as possible [14]. 

Almost 10% of patients may require invasive mechanical ventilation. Endotracheal intubation should be applied by a trained and most experienced physician with a rapid sequential intubation protocol. Intubation should be performed with video-laryngoscope, if possible. Intubation with flexible bronchoscopy can be used in difficult intubation. However, bronchoscopy is a high-risk procedure for aerolisation. Use of bag-mask ventilation should be avoided during preoxygenation. Preoxygenation could be performed with nonrebreather mask with reservoir. If bag-mask would be used, a filter should be attached to the bag mask. Neuromuscular blockers can be administered to suppress cough before intubation. Positive pressure ventilation should not be initiated before the endotracheal cuff is inflated and patients must be connected to mechanical ventilator directly without bag ventilation [15]. Closed system aspiration should be provided and bacterial/viral filters should be placed in both inspiratory and expiratory ports of the ventilator. For airway humidification, heat and moisture exchanger (HME) filter could be used. Unless absolutely necessary, bronchoscopic procedures should be avoided and metered dose inhaler (MDI) should be preferred instead of nebulization for bronchodilator therapy. However, due to mucus plugs, and increased resistance and dead-space ventilation, active heated humidifier (HH) can be preferred^7^7https://hsgm.saglik.gov.tr/depo/covid19/rehberler/COVID-19_Rehberi.pdf. 

In patients developing ARDS, low tidal volumes (4–6 mL/kg) and low inspiratory pressures (plateau pressure < 30 cm H2O, driving pressure which is plateau pressure minus positive end expiratory pressure < 14 cm H2O) should be applied. Deep sedation may be required to achieve target tidal volumes. In terms of low pH like < 7.15, tidal volume can be increased up to 8 mL/kg. Otherwise permissive hypercapnia may be allowed [16]. If there is no evidence of tissue hypoperfusion, conservative fluid support should be provided. PEEP titration should be applied at pressures that will prevent atelectotraumas and over distention. In moderate (PaO2/FiO2 < 200) and severe (PaO2/FiO2 < 100) ARDS patients, high PEEP may be applied instead of low PEEP. There is not enough data regarding recruitment manoeuvres. Even if the use of neuromuscular blocking agents is not routinely recommended, it can be applied in the presence of resistant hypoxemia or hypercapnia, despite sedation in moderate to severe ARDS with ventilator dyssnchrony and in the first 24–48 h of mechanical ventilation. In patients with PaO2/FiO2 < 150, more than 12 h of prone position could be applied daily in patients who are managed by conventional MV interventions. Prone position has been demonstrated to be helpful even in spontaneously breathing nonintubated patients, even with NIMV and HFNO. Inhaled nitric oxide (NO) administration could be used as a rescue therapy and could also theoretically be helpful to improve ventilation/perfusion match, since it has been speculated that perfusion is also impaired in ARDS due to COVID-19 and these patients have preserved compliance. Routine use of corticosteroids is not recommended [17]. Extracorporeal membrane oxygenation (ECMO) may be considered in patients with refractory hypoxemia despite lung-protective ventilation, and appropriate patients should be referred to experienced centres. Due to paucity of evidence for this virus and disease pathophysiology, probable advantages of ECMO is ambiguous. ECMO is not a therapy that should be considered in case of a major pandemic regarding the appropriate allocation of resources [18].

Dr. Gattinoni and his colleagues suggested that COVID-19 patients with respiratory failure have 2 phenotypes [19]. Phenotype characterized by low elastance (high compliance), so called Type L, has low ventilator perfusion ratio, low lung weight, and low recruitability, whereas Type H, characterized by high elastance (low compliance) has high right-to-left shunt, high lung weight, and high recruitability. Severe hypoxemia for high compliant lungs may be elucidated by the loss of lung perfusion regulation and hypoxic vasoconstriction. They recommend to increase FiO2 as a solution for hypoxemia for those who are not experiencing dyspnoea, in which Type L patient responds well. Early intubation may even cause transition to Type H phenotype. However, for intubated hypercapnic Type L patients, ventilation with more than 6 mL/kg up to 8–9 mL/kg does not bring risk of ventilator-associated lung injury. Since Type L phenotypes could have a tolerance in terms of mechanical power by the help of high compliance. Furthermore, administration of high PEEP for nonrecruitable lungs may cause hemodynamic impairment and fluid retention as well. Type H phenotype looks like usual severe ARDS. Therefore, higher PEEP, prone positioning, and even extracorporeal support are classical therapeutic choices. This suggestion has not been proved in large scale studies and postponing intubation in severe hypoxemia and increased work of breathing might be detrimental and early intubation in patients unresponsive to conventional O2 treatment and perhaps short course of NIMV and HFNO is recommended. 

During weaning from mechanical ventilation, patients under very low or no sedation should be evaluated for readiness as such, decreased O2 requirement (FiO2 < 0.40, PEEP < 8 cm H2O), hemodynamic stability, acceptable consciousness with preserved gag, and cough reflex. Rapid shallow breathing index could be checked so that patients with respiratory rate to tidal volume ratio < 100, have high likelihood of being successfully weaned from mechanical ventilation. Instead of weaning trial through t-piece, weaning from pressure support ventilation could be chosen due to less aerosol generation. In COVID-19 patients, high mucus plugs are being reported, therefore, to reduce the risk of reintubation timing of weaning and extubation should be individualized. Whether the cuff leak test should be performed routinely prior to extubation is unclear. If postextubation stridor is highly expected (female sex, fluid overload, prolonged intubation > 5–7 days, age > 80 years, large endotracheal tubes > 8–8.5 F, difficult and traumatic intubation), cuff-leak test could be performed weighing against risks and/or corticosteroids could be given during extubation. If possible, extubation should be performed in airborne isolation rooms with maximum PPE use. For postextubation respiratory failure, NIMV and/or HFNO should be applied very carefully in selected patients and if there is stridor, immediate intubation should be performed. For patients who fail weaning, a tracheostomy may be indicated which is considered to be a high-risk procedure for aerosolization [20]. 

Patients should receive pulmonary and extremity rehabilitation especially when they have low risk of infection transmission and meticulous nutritional support should be provided to prevent ICU-acquired weakness. 

## 6. Management of sepsis and shock

Sepsis is defined as organ failure due to dysregulated host response accompanying by suspected or documented infection. Organ failure symptoms and signs are; changes in consciousness, difficulty breathing, low oxygen saturation, decreased urine output, increased creatinine and heart rate, weak pulse, cold extremities or low blood pressure, signs of coagulopathy, thrombocytopenia, acidosis, increased lactate level or hyperbilirubinemia. Septic shock is thought in terms of fluid therapy resistant hypotension, vasopressor requirement and lactate level > 2 mmol/L to maintain mean arterial pressure (MAP) ≥ 65 mmHg. It should be remembered that myocarditis and associated arrhythmias, and cardiogenic shock may be seen in these patients [21]. 

Due to lack of evidence, most of the recommendations are weak or as best practice statements regarding management of COVID-19 patients with shock. It is recommended not to use static parameters in order to assess fluid responsiveness. Dynamic parameters like skin temperature, capillary refilling time, and/or serum lactate measurement are suggested. Conservative fluid management is superior to liberal approach for acute resuscitation. Buffered/balanced crystalloids are preferred over unbalanced in the initial treatment. Hydroxyethyl starch is not suggested which is a strong recommendation. In addition, gelatines and dextrans should not be used as well. Routine usage of albumin is not recommended. Norepinephrine is the first-line vasoactive agent. Vasopressin and then adrenalin should be added as a second-line agent, over titrating norepinephrine dose, if target MAP cannot be achieved by norepinephrine alone. Dopamine has no place but could be utilized if norepinephrine is not available as a last choice. Target MAP is 60–65 mmHg, rather than higher values. In spite of fluid resuscitation and norepinephrine, if patients have persistent cardiac dysfunction and hypoperfusion signs, dobutamine could be started^8^8https://www.sccm.org/getattachment/Disaster/SSC-COVID19-Critical-Care-Guidelines.pdf. 

First hour sepsis bundle should be implemented as baseline lactate measurement and repeated if first measurement is >2 mmol/L; blood cultures before antibiotics and antimicrobial therapy should be started within 1 h. If bacterial infection is suspected, appropriate empirical antimicrobial therapy should be initiated. The choice of antibiotic treatment is made according to the local epidemiological data and treatment guidelines of the patient’s clinical condition (community-acquired pneumonia, healthcare-related pneumonia, comorbidities, immunosuppression, health care admission in the last 3 months, prior antibiotic use). In intubated patients, diagnostic tests such as polymerase chain reaction (PCR) has higher sensitivity if it is done on tracheal aspirates rather than oro-nasal specimens^9^9WHO. Global surveillance for COVID-19 caused by human infection with COVID-19 virus: interim guidance. https://www.who.int/docs/default-source/coronaviruse/global-surveillance-for-covid-v-19-nal200321-rev.pdf (accessed March 22, 2020). For both corona PCR and bacterial cultures, tracheal aspirates should be preferred over bronchoscopic techniques. 

Although 30 mL/kg crystalloid (normal saline or ringer lactate) is suggested in the original bundle, due to the presence of ARDS fluid treatment should be individualized. Patients who are hemodynamically unstable, safety of airway should be provided by taking protective measures. Patients should not be transferred until stabilization especially of the airway. If the central catheter cannot be inserted, vasopressor treatment can be given through the possible widest vascular access. However, extravasation should be kept in mind^10^10https://hsgm.saglik.gov.tr/depo/covid19/rehberler/COVID-19_Rehberi.pdf. 

In the critically ill patients, venous thromboembolism prophylaxis should be undertaken due to immobility. However, in COVID-19 patients, presence of hypercoagulopathy is hypothesized and therefore, therapeutic anticoagulation and perhaps antiaggregant therapy should be considered in patients with no contraindications especially in patients with high d-dimer levels [22]. 

## 7. Cardiopulmonary resuscitation (CPR)

In case of cardiac arrest, CPR should be managed with as few people as possible with PPE which must be worn by all members of the team before entering the room. No chest compressions or airway procedures should be done without full PPE use. CPR should be initiated by only chest compressions which could also be performed by automatic resuscitators and preoxygenation could be provided with nonrebreather face masks with reservoir to prevent aerosol contamination. Recognising the shockable rhythms as soon as possible and appropriate interventions might maintain circulation and prevent the need of further respiratory support like intubation. If bag mask ventilation and tracheal intubation are needed, at least two physicians can apply this procedure by the help of oropharyngeal airway and video-laryngoscope. All procedures should be debriefed after CPR for personal safety check and patient clinical evaluation^11^11https://www.resus.org.uk/media/statements/resuscitation-council-uk-statements-on-covid-19-coronavirus-cpr-and-resuscitation/covidhealthcare/).

## 8. Radiology

In the differential diagnosis of COVID-19 pneumonia, imaging tests should also be used in addition to the patient’s history, clinical and laboratory findings and coronavirus specific diagnostic tests. Thorax computerised tomography (CT) examination can be useful in diagnosis and can provide important clues in the initial evaluation of novel coronavirus pneumonia. Multiple patched ground-glass opacities in bilateral lobular style, with peripheral location, are reported as characteristic thorax CT findings of COVID-19 pneumonia [23]. If CT is not applicable during ICU stay due to cardiorespiratory instability, lung ultrasound could be a surrogate imaging method to chest radiography or CT scanning being a highly sensitive and specific technique for the diagnosis and follow-up of these cases [24]. Ultrasound may also be used to make sure the proper placement of the endotracheal tube and diagnosis of complications such as pneumothorax [25]. 

## 9. Conclusion

In the light of the data obtained from COVID-19 pandemic and hospital follow-up of these critically ill patients, the needfulness of intensive care units with well-organized structure and trained HCW, has emerged once again. Intensive care science plays a locomotive role in this kind of outbreak management. The contribution of intensivists who are dealing with complex organ failures is very important in the training of healthcare professionals for outbreak planning. Providing the emotional support of HCW while organising these plans, increasing the motivation by using the available communication tools is crucial to prevent burnout by minimizing fear and anxiety [5].

## Acknowledgments

Akın Kaya is a member of COVID-19 Advisory Committee of Ministry of Health of Turkey. Arzu Topeli is a member of COVID-19 Advisory Committee of Ministry of Health of Turkey.
